# Supplementary material for: Alkaline earth metal vanadates as sodium-ion battery anodes
Source: Nat Commun. 2017 Sep 6;8:460. doi: 10.1038/s41467-017-00211-5 (PMC5587687; doi:10.1038/s41467-017-00211-5)
Supplement: Supplementary file 1 — Supplementary Information [file 41467_2017_211_MOESM1_ESM.pdf]

File name: Supplementary Information
Description: Supplementary Figures, Supplementary Tables and Supplementary Note 1

File name: Peer Review File
Description:

Supplementary Information

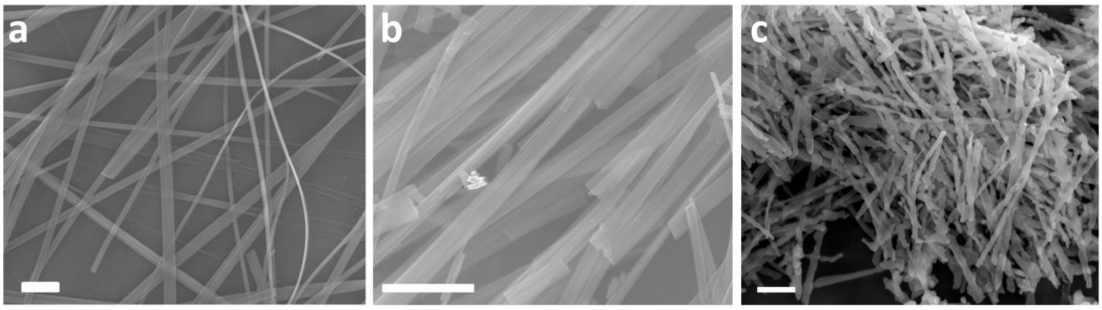

Supplementary Figure 1 | SEM images of the Ca-V-O nanowires. (**a**) Unsintered Ca-V-O sample, (**b**) CVO-450 and (**c**) CVO-550. The scale bars of (**a**, **b**, **c**) are 1 μm.

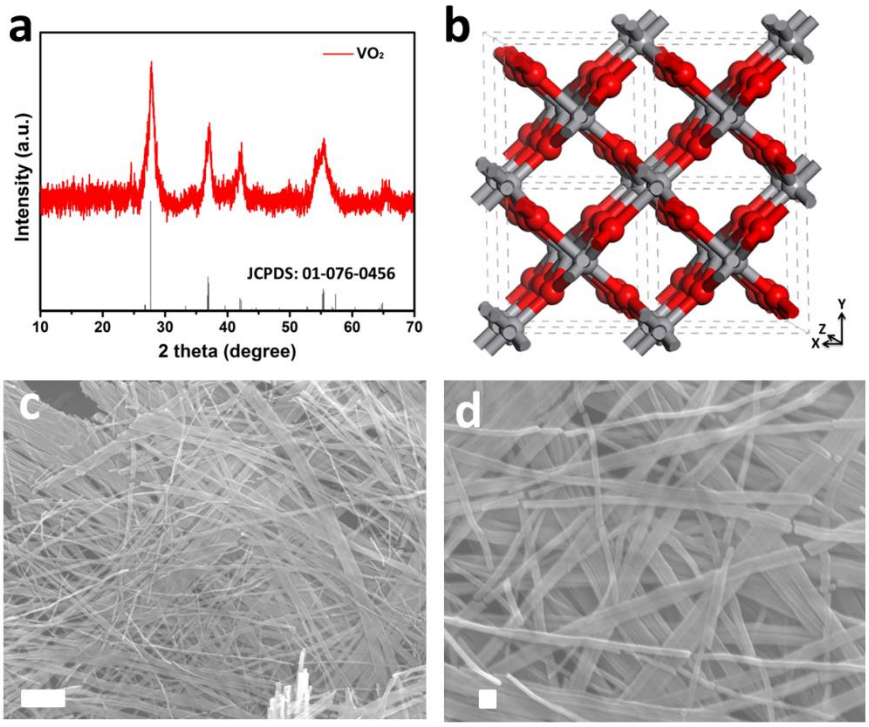

Supplementary Figure 2 | Characterization of VO_2-450 nanowires. (**a**) XRD pattern, (**b**) crystal structure and (**c,d**) SEM images of VO_2-450. The scale bar is 1 μm for (**c**) and 100 nm for (**d**).

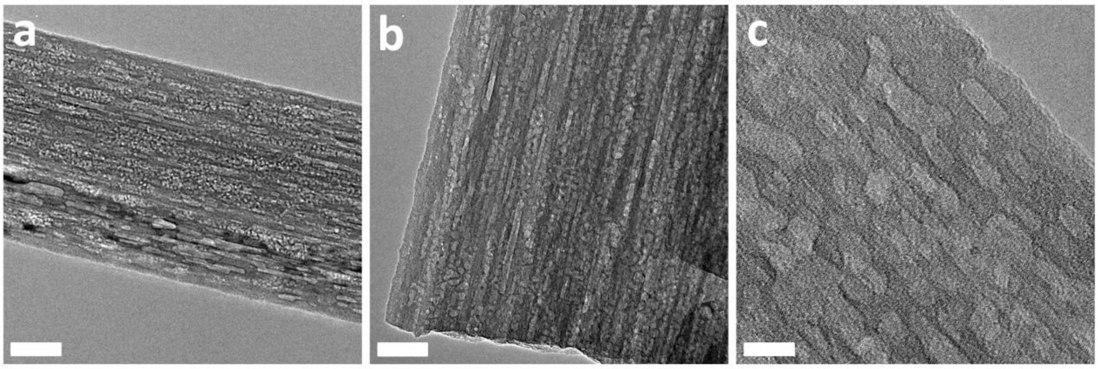

Supplementary Figure 3 | TEM images of CVO-450. The scale bars are 50 nm for (**a**, **b**) and 10 nm for (**c**).

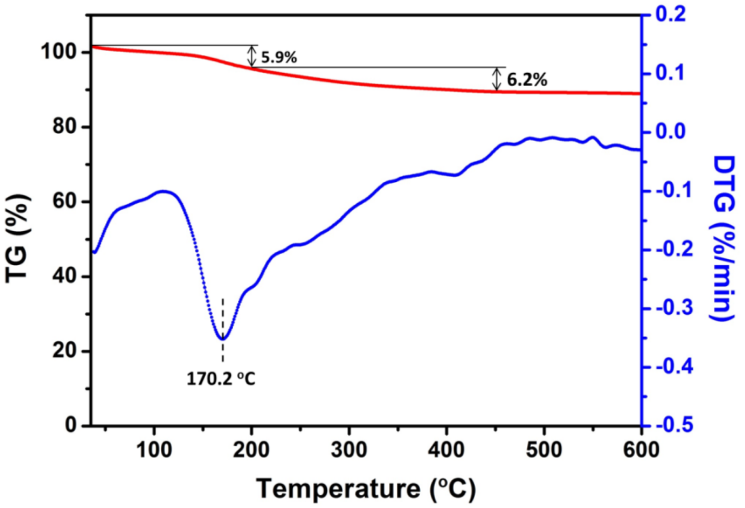

Supplementary Figure 4 | TG/DTG curves of unsintered Ca-V-O nanowire sample.

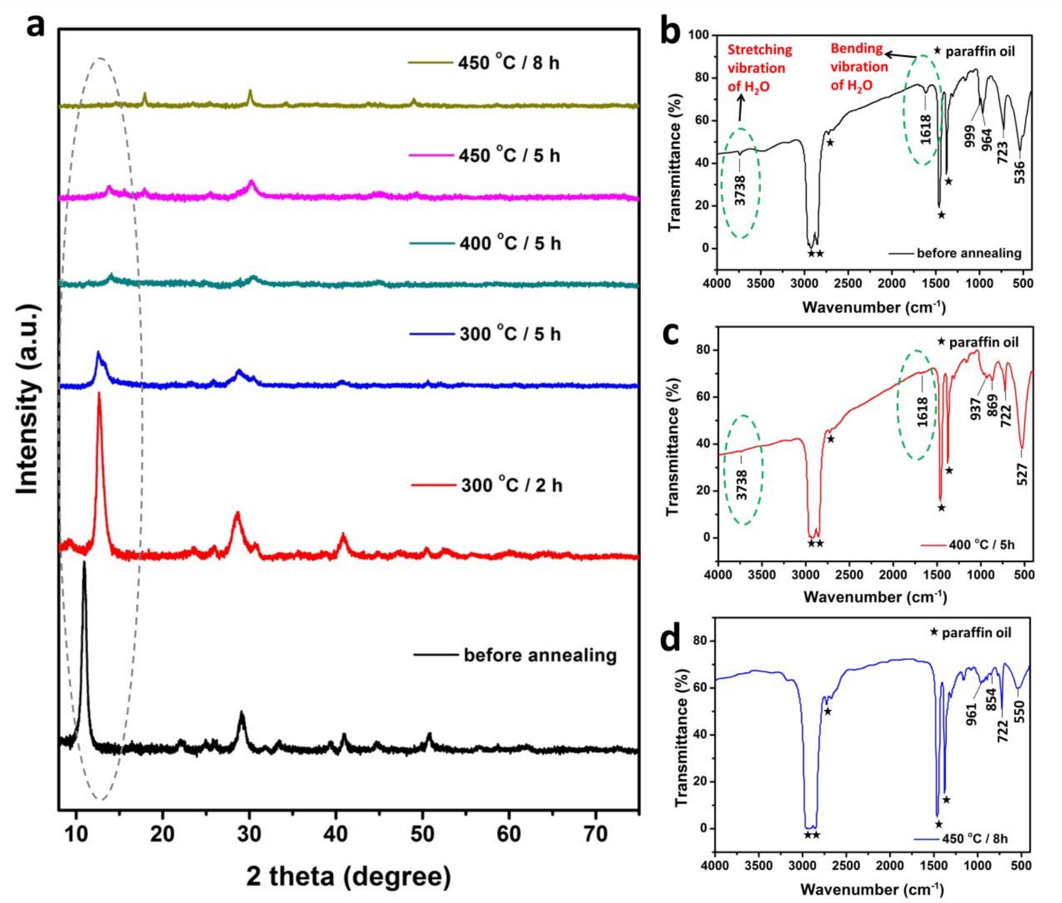

Supplementary Figure 5 | (a) XRD patterns of the Ca-V-O nanowire samples at different annealing condition. (b–d) FT-IR spectra of Ca-V-O nanowire samples before annealing (b), sintered at 400 ^{o}C for 5 h (c) and sintered at 450 ^{o}C for 8 h (d), respectively. (For the FT-IR spectra, the paraffin oil was used as the dispersant rather than KBr to avoid the moisture absorption during the test.)

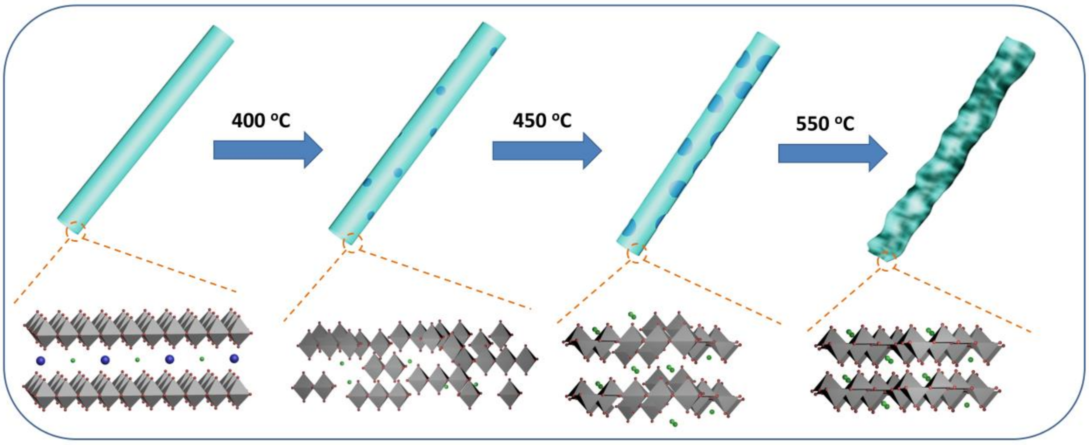

Supplementary Figure 6 | Illustration of the formation mechanism of the CVO-450 and CVO-550 nanowires.

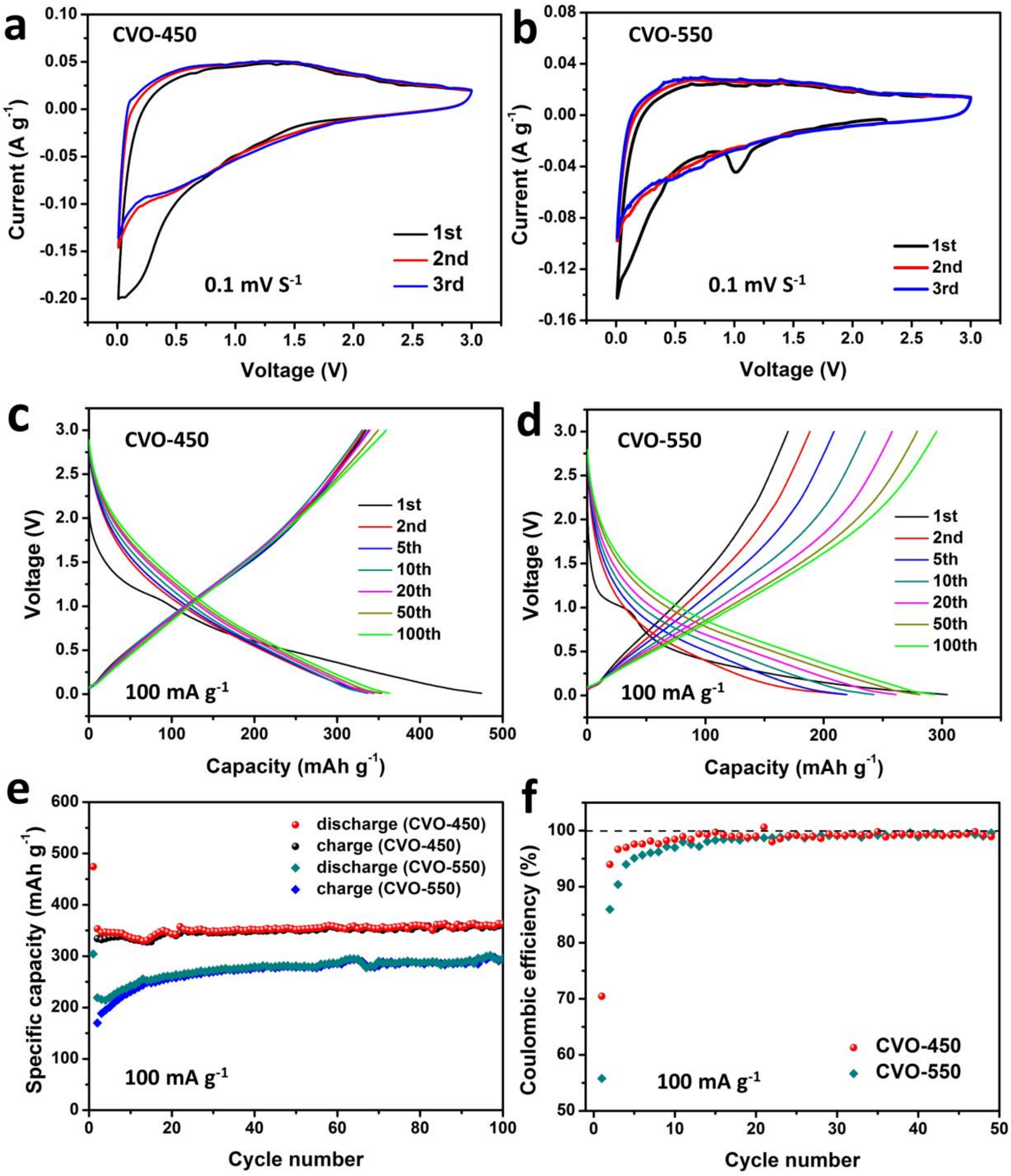

Supplementary Figure 7 | Electrochemical performance of CVO-450 and CVO-550. (**a,b**) CV curves of CVO-450 and CVO-550 at the scan rate of 0.1 mV s^{-1} in the voltage range from 0.01 to 3.0 V *versus* Na^+/Na. (**c,d**) Discharge-charge profiles of CVO-450 and CVO-550 after different cycles at 100 mA g^{-1}. (**e**) Cycling performance of CVO-450 and CVO-550 at current density of 100 mA g^{-1}. (**f**) Comparison of the Coulombic efficiency between CVO-450 and CVO-550 at current density of 100 mA g^{-1}.

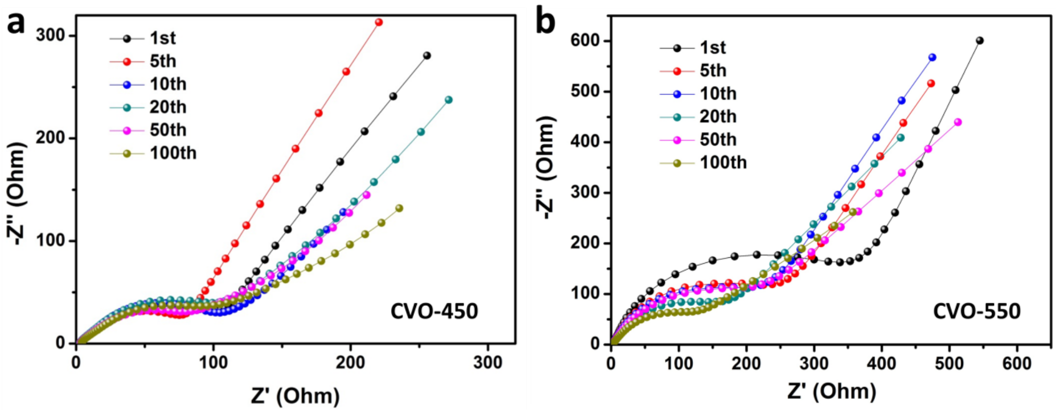

Supplementary Figure 8 | Nyquist plots of CVO-450 (a) and CVO-550 (b) after different cycles.

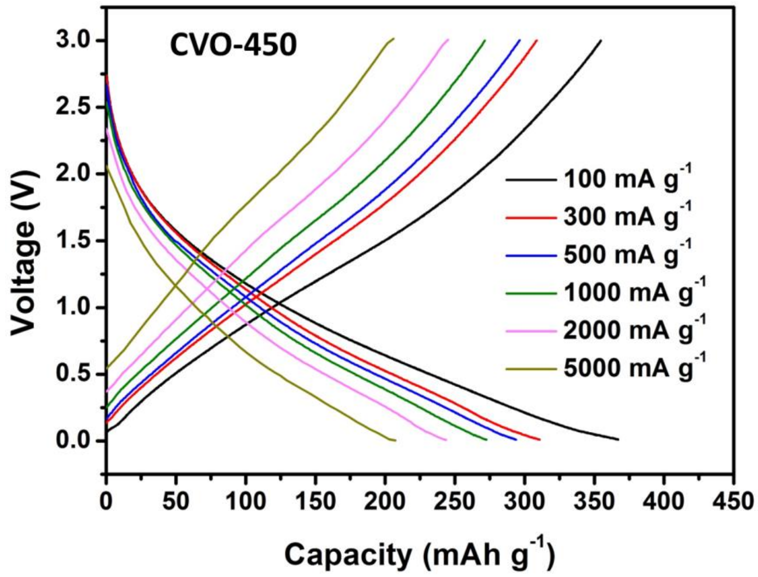

Supplementary Figure 9 | Discharge-charge curves of CVO-450 at 100, 300, 500, 1000, 2000, 5000 mA g^{-1}, respectively.

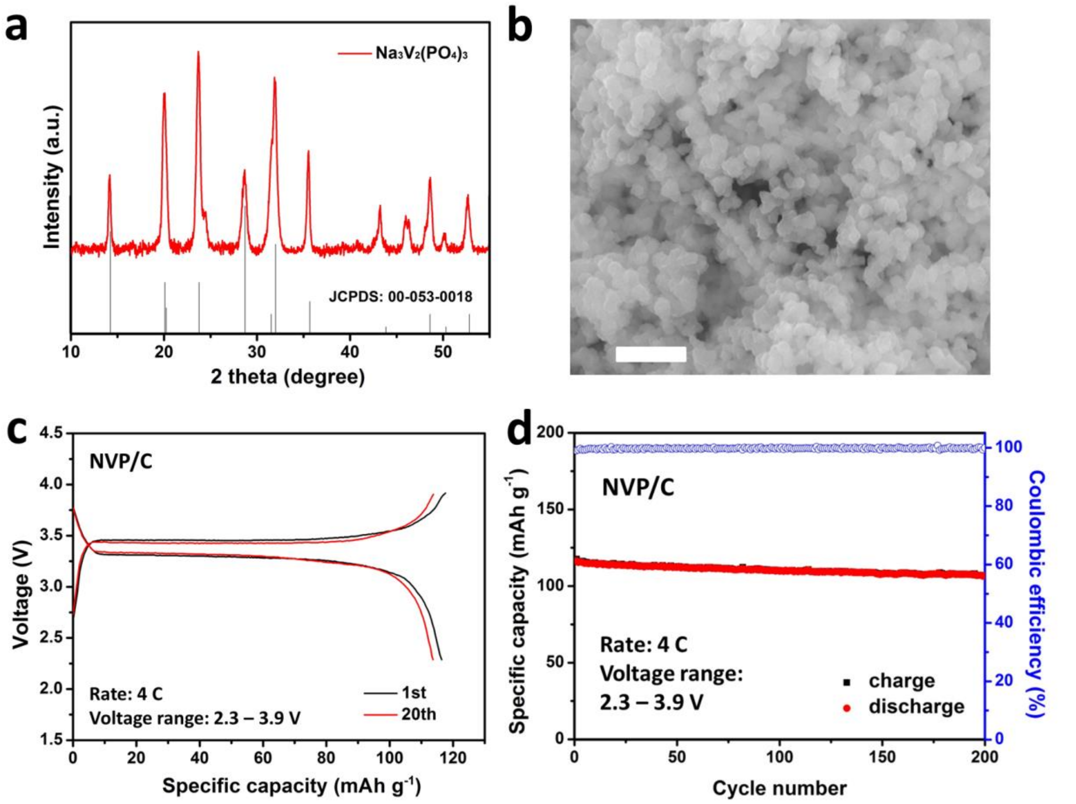

Supplementary Figure 10 | Characterization and electrochemical performance of $Na_3V_2(PO_4)_3$/C nanoparticles. (**a**) XRD pattern and (**b**) SEM image of $Na_3V_2(PO_4)_3$/C nanoparticles. Scale bar, 1 μm. (**c**) Charge-discharge profiles and (**d**) cycling performance of $Na_3V_2(PO_4)_3$/C nanoparticles at current rate of 4 C.

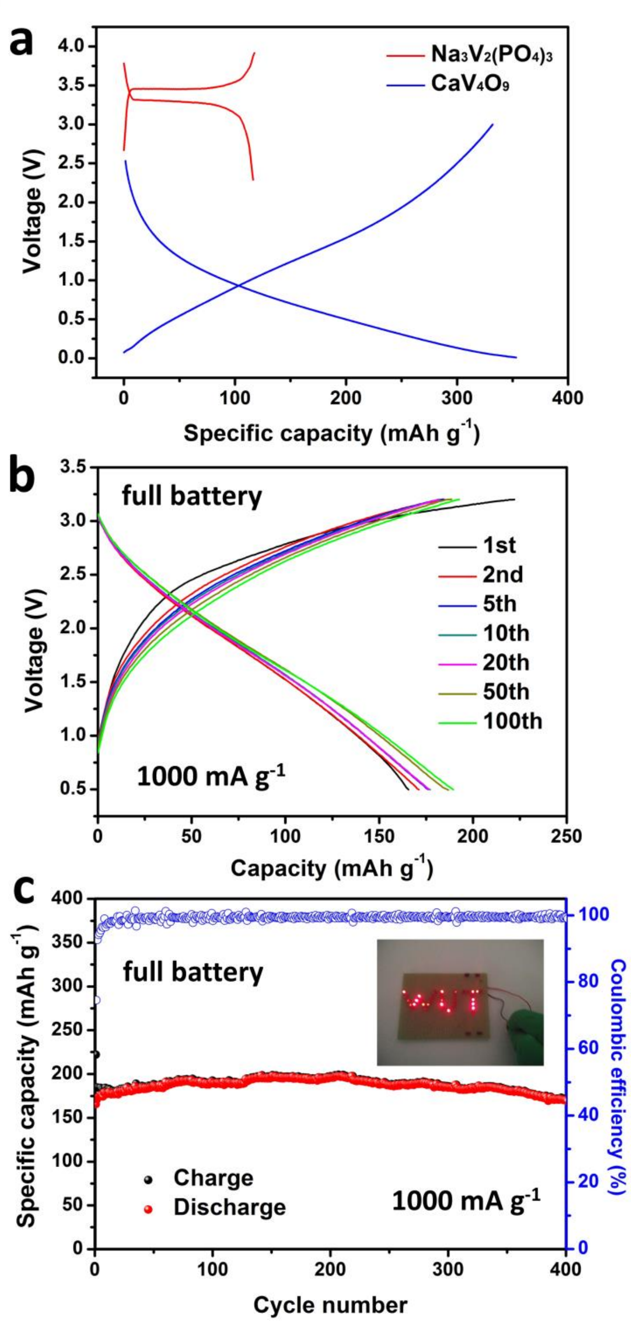

Supplementary Figure 11 | Electrochemical performance of $Na_3V_2(PO_4)_3$// CaV_4O_9 full battery. (**a**) Typical charge-discharge curves of $Na_3V_2(PO_4)_3$ and CaV_4O_9. (**b**) Charge-discharge curves of the full cell at the voltage range from 0.5 to 3.2 V after different cycles. (**c**) Cycling performance of the full cell at current density of 1000 mA g^{-1} based on the mass of CVO-450, the inset is the photograph of LED powered by the $Na_3V_2(PO_4)_3$// CaV_4O_9 full cells.

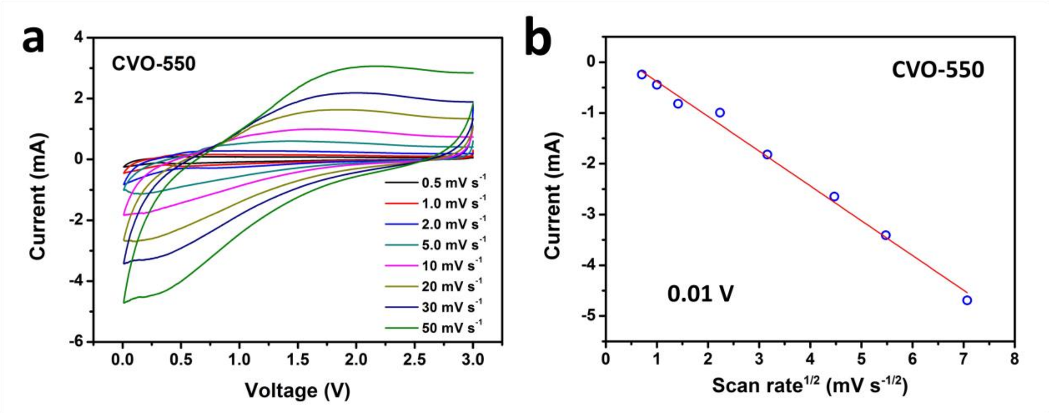

Supplementary Figure 12 | Cyclic voltammetry measurement of CVO-550 at different scan rates. (**a**) CV curves of CVO-550 at the scan rate from 0.5 mV s^{-1} to 50 mV s^{-1}. (**b**) The relation between the square root of the scan rate ($v^{1/2}$) and the corresponding currents at 0.01 V.

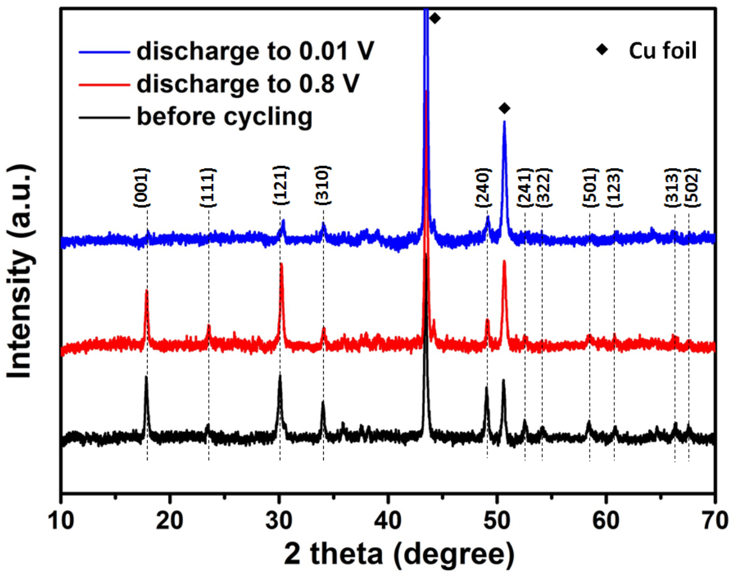

Supplementary Figure 13 | *Ex situ* XRD results of the CVO-550.

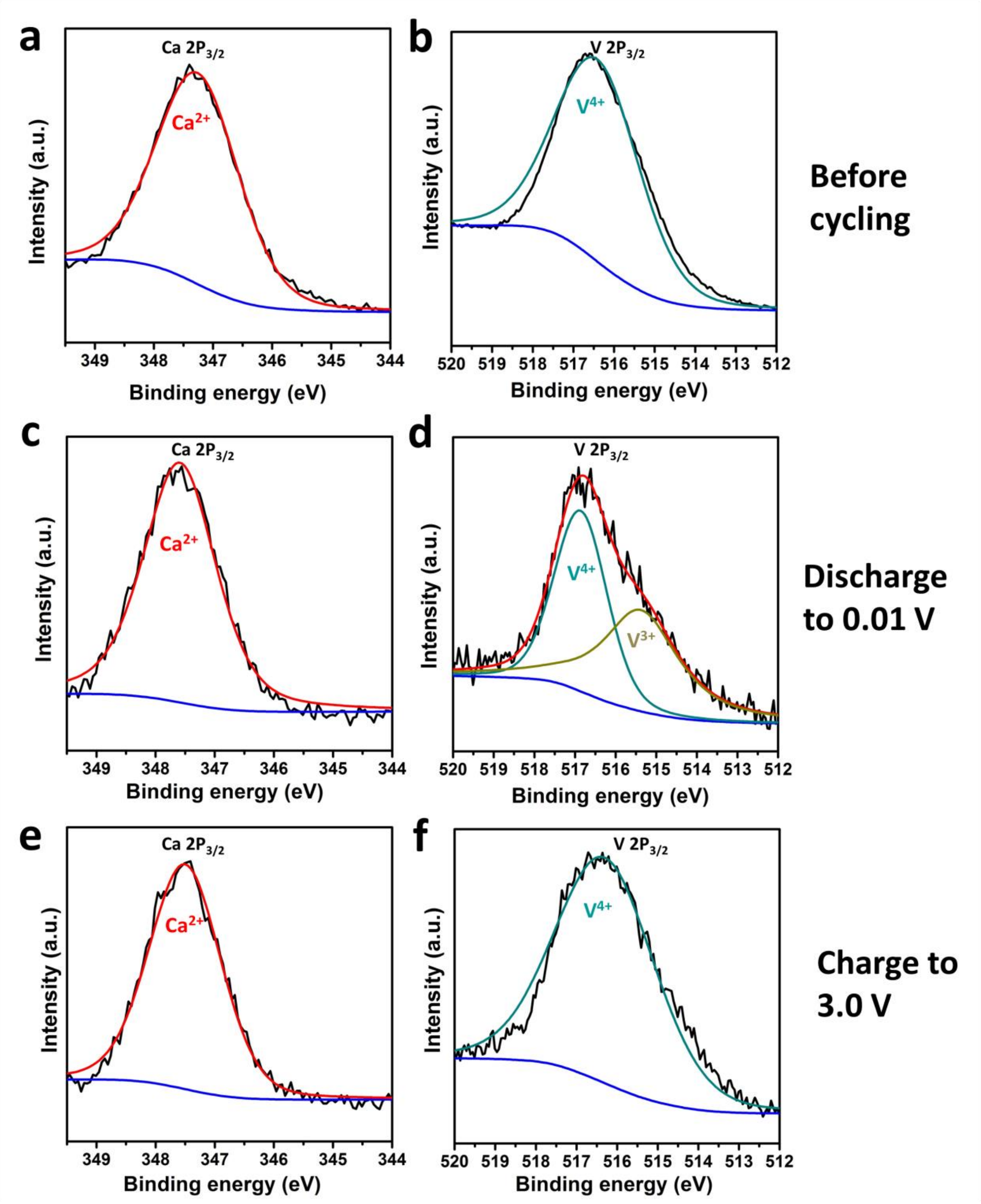

Supplementary Figure 14 | X-ray photoelectron spectra of Ca 2p$_{3/2}$ and V 2p$_{3/2}$ of CVO-450. (**a,b**) Before cycling, (**c,d**) discharge to 0.01 V, (**e,f**) charge to 3.0 V.

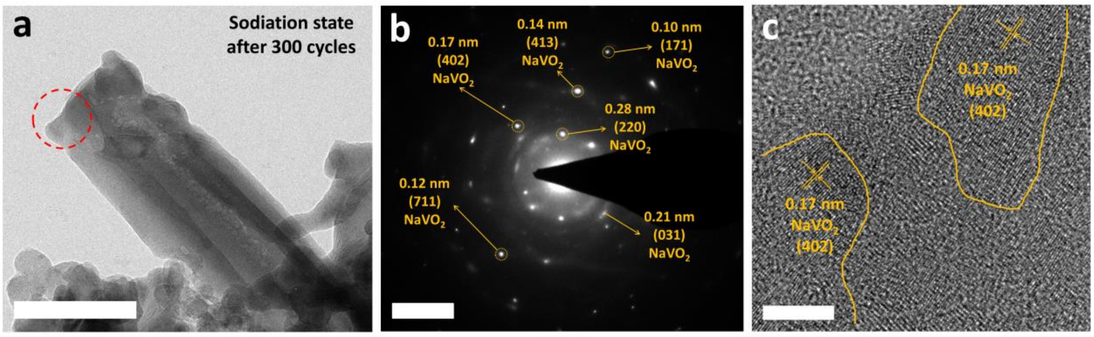

Supplementary Figure 15 | *Ex situ* TEM characterization of VO_2-450 at the sodiation state after 300 cycles. (**a**) TEM image. Scale bar, 200 nm. (**b**) SAED pattern. Scale bar, 5 1/nm. (**c**) HRTEM image. Scale bar, 5 nm.

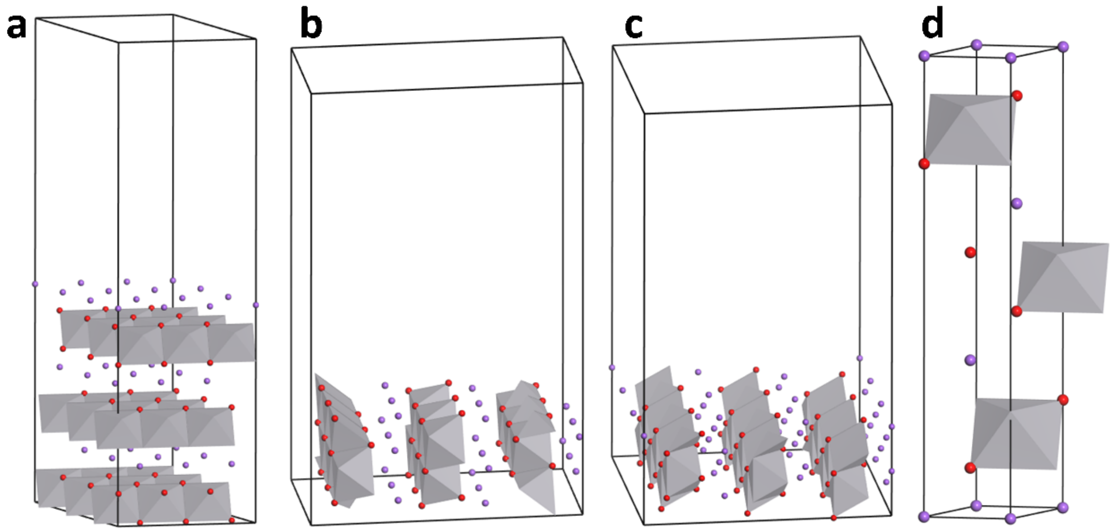

Supplementary Figure 16 | Models of $NaVO_2$ applied in DFT calculations. (**a-c**) Cells of $NaVO_2$ (001), (010) and (110) surfaces, respectively. (**d**) Cell of $NaVO_2$ crystal. The grey octahedrons with red balls indicate the VO_2 structure, and the violet balls denote the Na atoms.

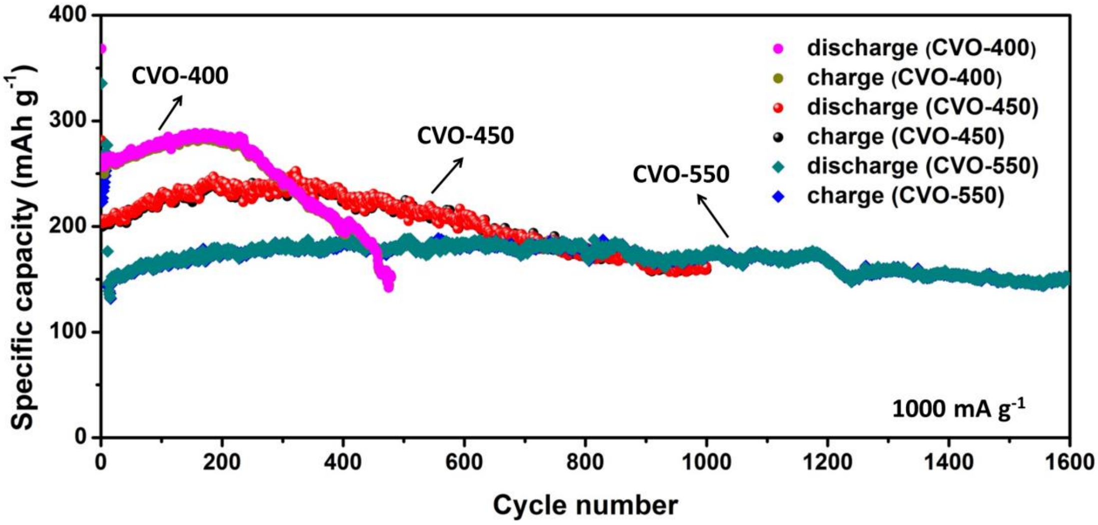

Supplementary Figure 17 | Comparison of specific capacity and cycling stability of CVO-400, CVO-450 and CVO-550 at current density of 1000 mA g^{-1}.

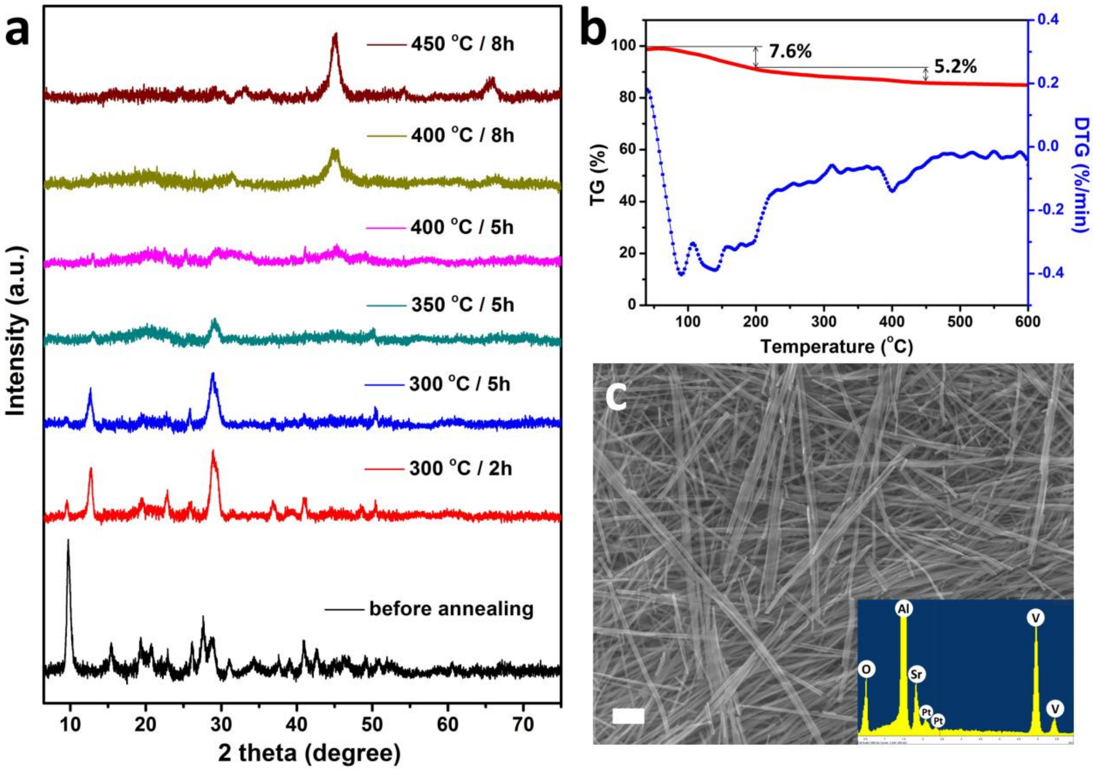

Supplementary Figure 18 | Characterization of the Sr-V-O nanowires. (**a**) XRD patterns of the Sr-V-O nanowire samples at different annealing condition. (**b**) TG/DTG curves of unsintered Sr-V-O nanowire sample at Ar atmosphere. (**c**) SEM image of the SVO-450. Scale bar, 1 μm. Inset is the EDS result of SVO-450.

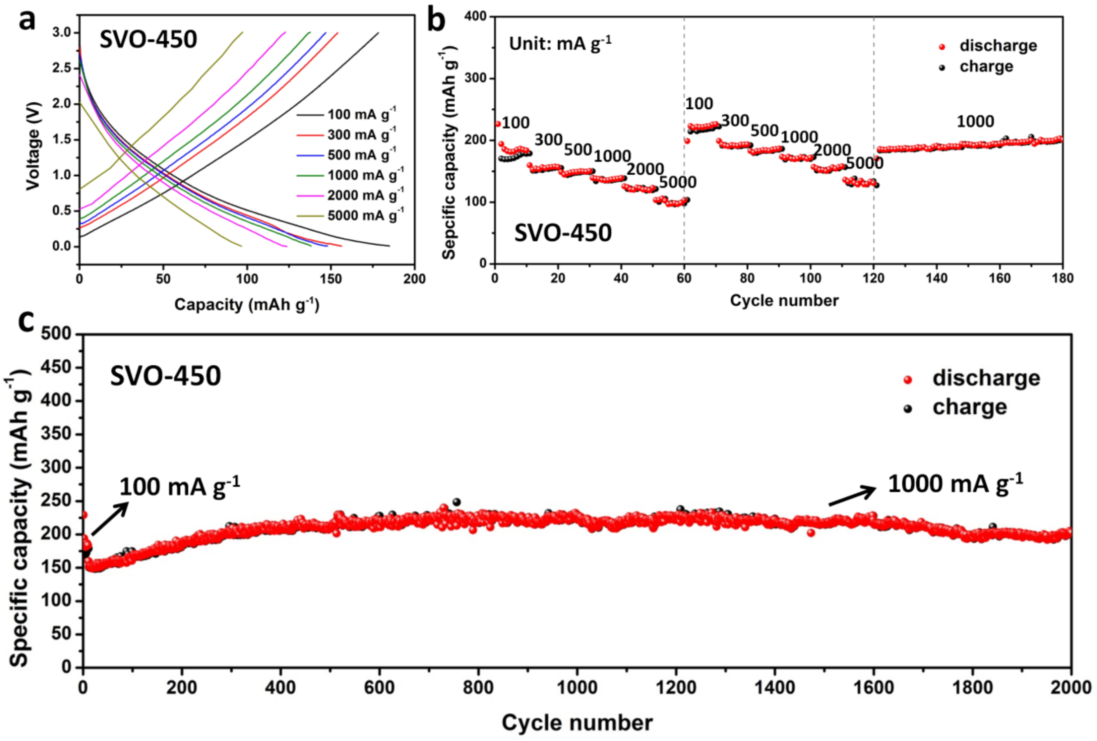

Supplementary Figure 19 | Electrochemical performance of SVO-450. (**a**) Discharge/charge profiles of SVO-450 at different current density. (**b**) Rate capability of the SVO-450. (**c**) Cycling performance of the SVO-450.

Supplementary Table 1 | The details of average capacity of CVO-450, CVO-550 and VO_2-450 at different current density.

CVO-450				CVO-550				VO_2-450			
Current density (mA g^{-1})	Average capacity (mAh g^{-1})			Current density (mA g^{-1})	Average capacity (mAh g^{-1})			Current density (mA g^{-1})	Average capacity (mAh g^{-1})		
	Stage I	Stage II	Stage III		Stage I	Stage II	Stage III		Stage I	Stage II	Stage III
100	363.9	363.9		100	265.2	331.5		100	234.8	251.1	
300	312.3	320.1		300	250.5	282		300	206.6	218.7	
500	298	305		500	225.3	253.2		500	200.7	202.1	
1000	273.6	279.5	291.7	1000	183.2	213.5	229.4	1000	188.4	170.4	161.3
2000	245.2	253.8		2000	146.1	173.8		2000	170.5	138.2	
5000	205	206.1		5000	103.4	130.2		5000	135.3	89.9	

Supplementary Table 2 | The peak list of CaV_4O_9 (JCPDS: 01-070-4469)

No.	h	k	l	d [Å]	2 Theta [deg]	Intensity [%]
1	1	1	0	5.88808	15.034	0.1
2	0	0	1	5.013	17.678	56.9
3	1	0	1	4.29478	20.665	0.6
4	2	0	0	4.1635	21.324	1.9
5	1	1	1	3.817	23.285	13.8
6	2	0	1	3.20288	27.832	6.8
7	1	2	1	2.98937	29.865	100
8	2	2	0	2.94404	30.336	6.4
9	3	1	0	2.63323	34.019	41
10	2	2	1	2.53863	35.328	1.7
11	0	0	2	2.5065	35.796	3.8
12	3	0	1	2.42829	36.99	0.1
13	1	0	2	2.40012	37.44	5.1
14	1	3	1	2.33119	38.59	1.7
15	1	1	2	2.30624	39.024	0.7
16	2	0	2	2.14739	42.043	0.9
17	2	3	1	2.09759	43.09	2.5
18	1	2	2	2.07936	43.487	18.3
19	3	3	0	1.96269	46.217	0.2
20	4	0	1	1.92257	47.239	1.1
21	2	2	2	1.9085	47.609	1.1
22	4	1	1	1.87329	48.561	2.4
23	2	4	0	1.86197	48.875	30.7
24	3	3	1	1.82761	49.856	0.6
25	3	1	2	1.81551	50.211	2.8
26	2	4	1	1.74546	52.376	11.1
27	3	2	2	1.69844	53.941	4.7
28	0	0	3	1.671	54.901	0.4
29	1	0	3	1.63834	56.091	0.7
30	1	5	0	1.63306	56.288	0.4
31	1	1	3	1.60752	57.264	1.2
32	4	0	2	1.60144	57.502	0.7
33	5	0	1	1.58047	58.338	16.5
34	4	1	2	1.57262	58.657	4.6
35	1	5	1	1.55275	59.483	2.1
36	2	0	3	1.55076	59.567	2.1
37	3	3	2	1.54531	59.798	1.1
38	1	2	3	1.52455	60.698	7.5
39	2	4	2	1.49469	62.043	0.7
40	5	2	1	1.47759	62.842	0.8
41	4	4	0	1.47202	63.107	0.4

42	2	2	3	1.45323	64.019	2.3
43	3	0	3	1.43159	65.105	0.7
44	5	3	0	1.42807	65.286	2.2
45	3	1	3	1.4109	66.181	6.7
46	5	0	2	1.38713	67.465	7.5
47	5	3	1	1.37343	68.23	1
48	3	2	3	1.3538	69.36	1
49	6	0	1	1.33752	70.328	0.4

Supplementary Table 3 | The peak list of $NaVO_2$ (JCPDS: 00-027-0825)

No.	h	k	l	d [Å]	2 Theta [deg]	Intensity [%]
1	0	0	3	5.44	16.281	55
2	0	0	6	2.714	32.977	15
3	1	0	1	2.564	34.967	12
4	1	0	2	2.468	36.373	20
5	1	0	4	2.183	41.325	100
6	1	0	5	2.025	44.716	2
7	1	0	7	1.734	52.749	12
8	1	0	8	1.603	57.441	20
9	1	1	0	1.498	61.891	15
10	1	1	3	1.445	64.428	5
11	1	0	10	1.378	67.973	5

Supplementary Table 4 | The peak list of CaO (JCPDS: 00-001-1160)

No.	h	k	l	d [Å]	2 Theta [deg]	Intensity [%]
1	1	1	1	2.76	32.412	40
2	2	0	0	2.39	37.604	100
3	2	2	0	1.69	54.233	63
4	3	1	1	1.45	64.179	20
5	2	2	2	1.38	67.861	20
6	4	0	0	1.2	79.87	10
7	3	3	1	1.1	88.898	7
8	4	2	0	1.07	92.094	25

Supplementary Table 5 | The peak list of VO_2 (JCPDS: 00-009-0142)

No.	h	k	l	d [Å]	2 Theta [deg]	Intensity [%]
1	-1	1	1	3.31	26.914	30
2	0	1	1	3.2	27.858	100
3	-1	0	2	2.68	33.408	30
4	-2	0	2	2.43	36.963	40
5	-2	1	1	2.422	37.089	60
6	2	0	0	2.418	37.153	30
7	-2	1	2	2.139	42.215	50
8	2	1	0	2.131	42.381	50
9	-1	2	1	2.048	44.187	10
10	0	2	1	2.022	44.786	30
11	-3	0	2	1.874	48.541	40
12	-1	2	2	1.727	52.979	20
13	-2	1	3	1.657	55.404	30
14	-2	2	2	1.654	55.514	30
15	2	1	1	1.65	55.66	60
16	-1	1	3	1.615	56.975	10

Supplementary Note 1.

The formation mechanism of low-crystalline structure with large amounts of cavities of the CVO-450 nanowires

To explore the formation mechanism of the low crystalline structure with large amounts cavities of the CVO-450 nanowires, TG/DTG analysis was performed on the unsintered nanowire samples at Ar atmosphere. The weight loss before 200 ^{o}C in the TG curve should be due to the evaporation of the physically absorbed water. From 200 to 450 ^{o}C, the weight loss was calculated as 6.2% (Supplementary Fig. 4), which was speculated to arise from the evaporation of crystal water in the samples.

XRD and FT-IR measurements were further performed on the samples after different sintering conditions (Supplementary Fig. 5). XRD results show that the strong peak at about 11° for the unsintered samples vanished gradually as the sintering temperature increase. Notably, the sample prepared at 400 ^{o}C is basically an amorphous state. When the temperature increases to 450 ^{o}C for 8h, the sample recrystallizes and forms low crystalline CaV_4O_9 (CVO-450). From FT-IR spectra, the stretching vibration and bending vibration of H_2O were clearly observed for the sample before annealing, but disappeared for the annealed sample at 450 ^{o}C for 8 h, consistent well with the XRD results. Based on the TG/DTG, XRD and FT-IR results, the formation mechanism of the low crystalline structure together with the cavities of CVO-450 nanowires was proposed as illustrated in Supplementary Fig. 6. The unsintered sample is a layered structure with the Ca^{2+} and H_2O molecules distributed in the interlayers. As the sintering temperature increases, the H_2O molecules gradually evaporate, which leads to the formation of cavities. Meanwhile, the loss of H_2O molecules in the interlayers results in the collapse of the layered structure and the decrease of the crystallinity. When the sintering condition was set at 450 ^{o}C for 8h, almost all the H_2O molecules evaporate and the structure recrystallizes to form low crystalline CaV_4O_9 nanowires (CVO-450). When the temperature increases to 550 $^{\circ}$C, the higher temperature push the diffusion of the atoms (mass transfer process) to form a stable crystal phase, leading to the higher crystallinity and the closure of the cavities together with the irregular morphology of CVO-550.
